# Invasive pneumococcal disease in hospitalised children from Lima, Peru before and after introduction of the 7-valent conjugated vaccine

**DOI:** 10.1017/S0950268819000037

**Published:** 2019-02-22

**Authors:** A. Luna-Muschi, F. Castillo-Tokumori, M. P. Deza, E. H. Mercado, M. Egoavil, K. Sedano, M. E. Castillo, I. Reyes, E. Chaparro, R. Hernández, W. Silva, O. Del Aguila, F. Campos, A. Saenz, T. J. Ochoa

**Affiliations:** 1 Universidad Peruana Cayetano Heredia, Lima, Peru; 2 Grupo Peruano de Investigación en Neumococo (GPIN), Lima, Peru; 3 Instituto Nacional de Salud del Niño, Lima, Peru; 4 Hospital de Emergencias Pediatricas, Lima, Peru; 5 Hospital Nacional Cayetano Heredia, Lima, Peru; 6 Hospital Edgardo Rebagliati, Lima, Peru; 7 Hospital Nacional Docente Madre Niño San Bartolome, Lima, Peru; 8 Hospital Daniel A. Carrion, Lima, Peru; 9 University of Texas Health Science Center at Houston School of Public Health, Houston, Texas, USA

**Keywords:** PCV7, Peru, pneumococcal conjugated vaccine, pneumococcal infections, *Streptococcus pneumoniae*

## Abstract

The objective of this study was to determine the serotype distribution and antibiotic resistance of invasive pneumococcal disease (IPD) strains in children from Lima, Peru, before and after the introduction of the 7-valent pneumococcal conjugate vaccine (PCV7), which was introduced in the national immunisation program on 2009. We conducted a prospective, multicentre, passive surveillance IPD study during 2006–2008 and 2009–2011, before and right after the introduction of PCV7 in Peru. The study was performed in 11 hospitals and five private laboratories in Lima, Peru, in patients <18 years old, with sterile site cultures yielding *Streptococcus pneumoniae*. In total 159 *S. pneumoniae* isolates were recovered. There was a decrease in the incidence of IPD in children <2 years old after the introduction of PCV7 (18.4/100 000 *vs.* 5.1/100 000, *P* = 0.004). Meningitis cases decreased significantly in the second period (*P* = 0.036) as well as the overall case fatality rate (*P* = 0.025), including a decreased case fatality rate of pneumonia (16.3% to 0%, *P* = 0.04). PCV7 serotypes showed a downward trend. Vaccine-preventable serotypes caused 78.9% of IPD cases, mainly 14, 6B, 5, 19F and 23F. A non-significant increase in erythromycin resistance was reported. Our findings suggest that the introduction of PCV7 led to a significant decrease of IPD in children under 2 years old and in the overall case fatality rate.

## Introduction

It is estimated that around half a million children under 5 years of age die every year in the world due to pneumococcal disease, primarily in developing countries [1]. Furthermore, antibiotic-resistant *Streptococcus pneumoniae* is a clinical and public health problem around the world. In Latin America, penicillin resistance is found in approximately 50% of isolates, with significant variations among countries [2].

Introduction of pneumococcal conjugate vaccines around the world has led to substantial reduction of invasive pneumococcal disease (IPD) among vaccinated and unvaccinated children due to the decreases in nasopharyngeal colonisation and transmission of vaccine-type pneumococci (herd effect) [3, 4]. On 2009, the 7-valent pneumococcal conjugated vaccine (PCV7) was implemented as part of the Peruvian national immunisation program in a 2 + 1 dose schedule. PCV10 was introduced in 2011 and PCV13 was introduced in 2015.

Surveillance of serotypes is required to measure the impact of vaccination. Knowledge of local and regional patterns of serotype distribution and antibiotic resistance is essential for developing effective strategies for vaccination and treatment protocols both for children and adults. The aim of this study was to compare the incidence of IPD, *S. pneumoniae* serotype distribution and antibiotic resistance patterns before (2006–2008) and after (2009–2011) PCV7 introduction in Lima, Peru.

## Material and methods

### Study design

This study was conducted during 2 periods: May 2006–April 2008, as part of a previously published study [5] and during July 2009–June 2011; each consisting of 2-year prospective, passive, multicentre surveillance studies of IPD in 11 public hospitals and five private clinical laboratories in Lima. Cultures were ordered by the attending physicians based on clinical judgment and the hospital's protocol. We followed the same surveillance methodology in both study periods; the pediatrician from each participating hospital, member of our research group, reviewed all positive cultures for *S. pneumoniae* at the hospital´s microbiology laboratory daily. There was no specific sampling, we included all positive cultures from sterile sites during the surveillance period. The study was approved by the Institutional Review Board of Universidad Peruana Cayetano Heredia (Lima, Peru) and by each participating hospital's board.

### Case definition

Invasive pneumococcal disease cases were defined by isolation of *S. pneumoniae* from normally sterile sites (blood, cerebral spinal fluid (CSF), pleural fluid, joint fluid, or peritoneal fluid) from patients <18 years of age, admitted at the participating hospitals. *S. pneumoniae* isolates from non-sterile (nasopharynx, pharynx, tonsils, or sputum) or unknown sites were excluded. Clinical and epidemiological data were obtained from the medical charts.

The diagnosis of pneumonia, meningitis, sepsis and other diagnoses were taken from medical records. Pneumococcal pneumonia was defined by a positive blood or pleural fluid culture in the presence of an infectious process with fever and respiratory distress and evidence of pulmonary infiltrates on the chest X-ray. Pneumococcal meningitis was defined by isolation of *S. pneumoniae* in CSF or a positive blood culture in the presence of signs and symptoms of neurological involvement. Pneumococcal sepsis was defined by a positive blood culture in the presence of a systemic inflammatory response syndrome (SIRS).

### Incidence rate determination

We determined the populations served by each hospital in each district of the Province of Lima using an estimate based on each district's population and the number of hospitals and clinics available. It was estimated that 75% of the total pediatric population is served by the study's participating hospitals and clinics. The mean annual IPD rate (number of cases/100 000 children) was calculated based on rates in the study's two periods among children <2 years of age (1 month–24 months of age) and <5 years of age (1 month–60 months of age). The population of Lima was obtained from the National Institute of Statistics and Informatics (INEI). During the first study period, the city of Lima had a mean population of 249 799 children <24 months of age and 773 280 children <60 months of age and during the second study period, a population of 316 778 and 791 331.5, respectively.

### Laboratory studies

We transported *S. pneumoniae* isolates in the blood agar plate, from each hospital to a central laboratory on the day of isolation. *S. pneumoniae* identification was confirmed by conventional microbiology methods based on the colony morphology, alpha hemolysis, Gram stain, bile solubility and optochin susceptibility. We used Etest^®^ (AB Biodisk, Solna, Sweden) to determine antimicrobial susceptibility by minimal inhibitory concentration (MIC) to four antibiotics: ceftriaxone, chloramphenicol, erythromycin and penicillin. Susceptibility was assessed according to the 2017 performance standards of the Clinical and Laboratory Standards Institute (CLSI) [6]. Serotyping was performed by Quellung reaction at the Center for Disease Control and Prevention (CDC), Atlanta, USA.

### Statistical analysis

The data are presented as number of cases and percentages. We compared continuous variables between two sub-groups with *t* tests if normally distributed and with Wilcoxon rank-sum tests if not normally distributed and compared dichotomous or categorical values between two subgroups with *χ*^2^ tests. When comparing small values, we used Fisher's exact test, to approximate a better estimate.

## Results

A total of 159 *S. pneumoniae* isolates were recovered during both study periods, 101 from 2006 to 2008 and 58 from 2009 to 2011. Patients’ age ranged from 1 month to 15.4 years with a median of 14 months in the first period and 29 months in the second period. The main diagnoses in both periods were pneumonia and meningitis (Table 1). Pneumococci were isolated mainly from blood and cerebrospinal fluid. The percentage of meningoencephalitis cases significantly decreased from 38.6% in the first period to 22.4% in the second (*P* = 0.036).

**Table 1.** General characteristics of children with invasive pneumococcal disease before and after PCV introduction

	2006–2008	2009–2011
	*n* = 101	*n* = 58
Age, mean (IQR)	1.17 (0.75–2.88)	2.5 (1.17–7.08)
<2 years, *n* (%)	69 (68.3)	24 (41.4)*
2–6 years, *n* (%)	19 (18.8)	14 (24.1)
6–18 years, *n* (%)	13 (12.9)	20 (34.5)*
Male sex, *n* (%)	59 (48.4)	30 (51.7)
Source of *S. pneumoniae* culture, *n* (%)		
Blood	59 (58.4)	37 (63.8)
Cerebrospinal fluid	29 (38.7)	11 (19.0)
Pleural fluid	7 (6.9)	5 (8.6)
Other	6 (5.9)	5 (8.6)
IPD medical condition, *n* (%)		
Pneumonia	48 (47.5)	30 (51.7)
Meningoencephalitis	39 (38.6)	13 (22.4)**
Sepsis-bacteremia	8 (7.9)	8 (13.8)
Other	3 (3.0)	7 (12.1)
Fatal cases, *n*/*N* (%)	20/91 (22.0)	4/53 (7.0)**
Pneumonia	7/43 (16.3)	0/29 (0.0)**
Meningitis	11/34 (32.3)	2/11 (18.2)
Other	2/8 (25)	2/13 (15.4)

IQR, interquartile range.

*P < 0.001, ** *P* < 0.05.

**Fig. 1.**
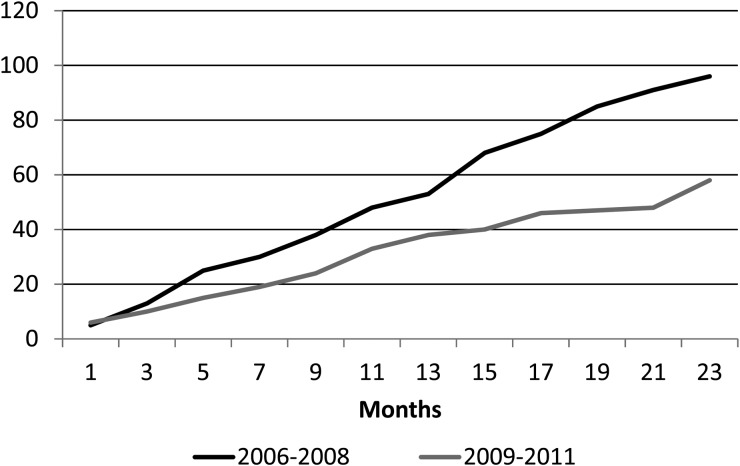
Cumulative cases of invasive pneumococcal disease (IPD) according to study period. Black line: 2006–2008; grey line 2009–2011.

Children under 2 years of age are the group with the highest risk for IPD. [5]. The incidence rate of IPD in children <2 years of age in Lima was 18.4/1 00 000 (17.1/1 00 000 in the first year and 19.8/100 000 in the second year) during the first period, showing a significant decrease during the second period, where IPD incidence was only 5.1/1 00 000 (7.2/1 00 000 in the first year and 2.9/1 00 000 in the second year (*P* = 0.004)). There was also a decrease in the incidence rate in children <5 years of age, although not significant. The cumulative cases of IPD before and right after PCV-7 introduction are shown in Figure 1.

The overall case fatality rate showed a significant decrease from 22.0% to 7.5% after vaccine introduction (*P* = 0.025). The fatality rate in children <2 years was 23.2% (16/52) and 13% (3/23) during the first and second period, respectively. The case fatality rate of pneumococcal pneumonia decreased significantly after vaccine introduction, *P* = 0.04. (Table 1).

The comparison of serotype distribution in 2006–2008 against 2009–2011 is summarised in Table 2. PCV7 serotypes represented 67.5% of isolates during the first period and 56.9% during the second period; the decrease was not significant. However, non-vaccine serotypes have shown a rise since PCV7 introduction, as they increased from 32.5% to 43.1% of IPD strains. When assessing for serotypes included in PCV13, we found that 78.9% of IPD cases would have been caused by vaccine-preventable serotypes. The most frequently isolated serotypes were serotypes 14, 6B, 5, 19F, 23F and 19A. During the second period, seven patients received at least one dose of PCV7 and two received PPV23.

**Table 2.** Serotype distribution of *Streptococcus pneumoniae* before and after PCV introduction

	2006–2008	2009–2011
Serotypes	*N* = 99	*N* = 58
	*n* (%)	*n* (%)
14	26 (26.3)	11 (19.0)
6B	20 (20.2)	6 (10.3)
19F	11 (11.1)	9 (15.5)
23F	6 (6.1)	6 (10.3)
4	1 (1.0)	0 (0.0)
18C	1 (1.0)	1 (1.7)
9 V	1 (1.0)	1 (1.7)
7F	0 (0.0)	0 (0.0)
5	6 (6.1)	2 (3.4)
1	0 (0.0)	2 (3.4)
19A	4 (4.0)	5 (8.6)
3	1 (1.0)	2 (5.2)
6A	4 (4.0)	0 (0.0)
Other	18 (18.2)^a^	12 (20.7)^b^
Vaccine serotypes		
PCV7	66 (67.5)	33 (56.9)
PCV10	72 (72.7)	37 (63.8)
PCV13	81 (81.8)	44 (75.9)

a Other serotypes: Non-typable (three strains); 11A, 15A, 25 (2 strains each), 9N, 12F, 13, 16F, 23A, 34, 38 (one strain each).

b Other serotypes: 10A (four strains); 7C,11A, 12F, 23A, 23B, 34, 35F, 38 (one strain each).

All 157 *S. pneumoniae* isolates were evaluated for antibiotic susceptibility (Table 3). Antibiotic resistance was high for penicillin in both periods; the highest resistance rates were among isolates recovered from cerebrospinal fluid (46.2% and 50%, respectively). We found a non-significant increase in resistance to erythromycin (24.8% *vs.* 37.5%). The most frequently resistant serotypes in were 14 (17/157), 19F (17/157) and 6B (12/157).

**Table 3.** Antimicrobial resistance of *Streptococcus pneumoniae* strains by minimal inhibitory concentration (MIC)

	2006–2008 (*n* = 101)			2009–2011 (*n* = 56)		
	Susceptible	Intermediate	Resistant	Susceptible	Intermediate	Resistant
	*N* (%)	*N* (%)	*N* (%)	*N* (%)	*N* (%)	*N* (%)
Penicillin^a^						
Meningitis	21 (53.8)	–	18 (46.2)	7 (50.0)	–	7 (50.0)
Non-meningitis	57 (91.9)	1 (1.6)	4 (6.5)	42 (100.0)	0 (0.0)	0 (0.0)
Ceftriaxone^a^						
Meningitis	30 (76.9)	8 (20.5)	1 (2.6)	10 (71.4)	4 (28.6)	0 (0.0)
Non-meningitis	59 (95.2)	1 (1.6)	2 (3.2)	38 (90.5)	2 (4.7)	2 (4.7)
Chloramphenicol^b^	89 (88.1)	–	12 (11.9)	49 (89.1)	–	6 (10.9)
Erythromycin	76 (75.2)	0 (0.0)	25 (24.8)	34 (60.7)	1 (1.8)	21 (37.5)

a In the first period 39 meningitis strains and 62 non-meningitis strains were analysed and in the second period 14 meningitis strains and 42 non-meningitis strains.

b In the second period 55 strains were analysed for chloramphenicol resistance.

## Discussion

In this prospective multicenter passive surveillance study in Peru, we found a significant decrease in IPD incidence in the post-vaccination period, especially among children <2 years of age, comparable with trends found in similar studies performed in Brazil and Uruguay [7, 8]. This decrease could be due to vaccine effect or a series of other factors that we may have not explored; such as, changes in population socio-economic status and healthcare access. On the other hand, limited laboratory resources could have also been an influencing factor. However, even though the incidence of PCV7 serotypes showed a downward trend, the decrease was not significant, opposing to what was found in the previously mentioned studies. This could be due to an unachieved herd effect, secondary to a vaccination rate of 45% reported in Peru during the first years of PCV7 introduction [9]. The most common serotypes were 14, 6B, 19F and 23F, similar to other countries in the region [10–12]. A previous study made by Hawkins *et al*. described serotypes and common PBP mutations by whole genome sequencing [13]. They identified the expansion of serotype 19F after PCV7 introduction; specifically, the 19F/ST1421 lineage, which predicted beta lactam resistance. The current study complements their findings, as it is focused in the epidemiological characteristics of children with IPD and the evaluation of resistance phenotype to other commonly used antibiotics. Almost 80% of IPD cases were caused by vaccine-preventable serotypes (PCV13). Therefore, higher vaccination coverage should lead to a significant reduction in the burden of disease in the following years.

In developing countries, non-bacteremic pneumonia causes most pneumococcal deaths in children [14, 15]. After vaccine introduction, there were no reported cases of death in children with a diagnosis of pneumonia compared with a case fatality of 14.6% in the pre-vaccination period. The case-fatality rate was similar in the second period to that of other Latin American countries such as Chile and Brazil [16, 17]. Even though there is a clear trend to decreased fatality, several factors, such as inadequate disease registration and suboptimal microbiological identification could have affected pneumococcal strains isolation and lack of PCV7 serotypes decline.

The increasing rate of penicillin resistance of *S. pneumoniae* in Latin America over the past years has raised concerns due to the risk of treatment failure and high healthcare costs [18]. Countries like Mexico and Venezuela have reported resistance rates over 80% [2]. Our results showed an elevated penicillin-resistance rate (46.2% and 50%) in meningeal strains and showed a non-significant decrease in resistance in non-meningeal strains after vaccine introduction. Continuous surveillance is needed in order to guide appropriate empiric treatment of more severe pneumococcal diseases, such as pediatric meningitis.

We found a non-significant increase in erythromycin resistance. Even though this is non-conclusive, we consider it shows an important trend that needs to be followed. For the last 30 years there has been a worldwide increase in *S. pneumoniae* resistance to macrolides [19–22]. This could render them ineffective in pneumococcal disease as recent studies suggest there is an increased risk of treatment failure of macrolides in respiratory tract infections [22].

The present study has some limitations. First, the data were collected from a passive surveillance study, which could underestimate the incidence of IPD, nevertheless is the starting point for future and more comprehensive studies. In order to have a larger sample size, we would have extended the study period or increase the number of sites. Second, we have not searched for IPD cases in other hospitals beyond the ones included in our study; which also contributes to incidence underestimation. Finally, the results must be considered preliminary, due to the short time after introduction of the vaccine and the low vaccination rates achieved in our country.

## Conclusions

The introduction of the PCV7 in Peru has led to a significant decrease of IPD in children <2 years old, with a reduction of PCV7 serotypes, although not significant. Ongoing surveillance of patients with IPD is needed to determine if the decrease in the incidence persists and expands to older populations and to establish the effect of vaccination on serotype distribution and antimicrobial resistance. We intend to conduct follow-up surveillance studies in order to evaluate the effects of PCV10 and PCV13.
